# Functional characterization of the IFN-γ protein in mediating antiviral defence against Newcastle disease Virus in *Larus ridibundus*

**DOI:** 10.3389/fcimb.2026.1919500

**Published:** 2026-08-11

**Authors:** Hua Chang, Feiyan Dai, Honglin Cheng, Yi Chen, Xiaotong Zhao, Shengjie Ren, Hongli Zhang, Bofang Duan, Xun Xiang, Gang Duan

**Affiliations:** 1 College of Veterinary Medicine, Yunnan Agricultural University, Kunming, China; 2 College of Foreign Languages, Yunnan Agricultural University, Kunming, China; 3 College of Animal Science and Technology, Yunnan Agricultural University, Kunming, China

**Keywords:** antiviral defence, IFN-γ, Larus ridibundus, migratory wild, Newcastle disease virus

## Abstract

**Introduction:**

Newcastle disease virus (NDV) can spread across regions via migratory wild birds, posing potential threats to livestock breeding and public health security. Systematic information on the genetic characteristics, immunoregulatory functions, and antiviral activity of IFN-γ in the migratory wild bird *Larus ridibundus* remains limited.

**Methods:**

In this study, the complete coding sequence of *L. ridibundus* IFN-γ was cloned and analyzed bioinformatically. A prokaryotic expression system was used to produce purified recombinant IFN-γ, and specific polyclonal antibodies were prepared for protein identification. Peripheral blood lymphocytes were exposed to graded NDV doses, serial concentrations of recombinant IFN-γ, or combined IFN-γ and NDV treatment. Quantitative real-time PCR was used to measure NDV NP transcripts and the transcription of TLR7, MyD88, IRF7, IFN-α, IFN-γ, and Mx.

**Results:**

NDV infection was associated with increased transcription of TLR7 pathway-related genes. Among the tested IFN-γ concentrations, 200 ng/mL produced the strongest TLR7 transcriptional response. IFN-γ intervention significantly reduced intracellular NDV NP transcript abundance, and this reduction coincided with lower transcription of TLR7 pathway-related genes under combined treatment.

**Discussion:**

These findings indicate that *L. ridibundus* IFN-γ has *in vitro* anti-NDV activity and that this activity is associated with transcriptional modulation of the TLR7-MyD88-IRF7 pathway. Direct pathway dependence remains to be established by pathway-inhibition experiments.

## 1 Introduction

Wild migratory birds act as natural reservoirs and transmission vectors of avian pathogens, facilitating the cross-regional and cross-species spread of viral diseases within ecosystems. *Larus ridibundus*, a migratory bird of the family Laridae, exhibits seasonal migration behavior and overwinters in Kunming every year, forming a human–gull coexistence ecosystem. During overwintering, *Larus ridibundus* come into frequent contact with local residents, domestic poultry and other wild birds, promoting the cross-host transmission of avian viruses (Djurdjević et al., 2025). Owing to their long-distance migration, large flocking populations and marked inter-individual variation in immune status, *Larus ridibundus* are prone to viral infection and pathogen carriage during migration and overwintering, posing persistent risks to poultry farming and public health.

Newcastle disease virus (NDV), also known as Avian orthoavulavirus 1, is an enveloped, negative-sense single-stranded RNA virus of the family Paramyxoviridae; its velogenic strains are highly virulent and cause substantial losses to avian ecology and the poultry industry (Yang et al., 2020). Epidemiological investigations have confirmed that *Larus ridibundus* serve as natural carriers of NDV: virus shed in their feces and respiratory secretions can contaminate water sources, the environment and feed, driving the cross-regional and cross-species spread of the virus (Chang et al., 2014, Chang et al., 2018). However, the molecular mechanism by which *Larus ridibundus* resist NDV infection remains unclear, which limits a comprehensive understanding of antiviral immunity in wild birds. Elucidating the key immune regulatory molecules and their antiviral mechanisms in this species therefore has both theoretical and practical significance.

Interferons (IFNs) are a family of key cytokines produced by host cells in response to immunogens, mitogens or other inducers (Vlachiotis and Andreakos, 2019; Cheng et al., 2015). Acting via autocrine and paracrine pathways, IFNs interact with diverse immune factors to form an immune regulatory network and participate in antiviral defense, inflammatory responses and immune homeostasis (Ramos et al., 2021). Interferon-γ (IFN-γ), the sole member of the type II IFN family, possesses immunoregulatory functions distinct from those of type I and type III IFNs (Samuel, 2020). Whereas type I IFNs mainly suppress viruses directly, IFN-γ bridges innate and adaptive immunity and exerts dual direct-antiviral and broad-spectrum immunomodulatory effects (Schroder et al., 2004; Goodbourn et al., 2000; Mesev et al., 2019; Adolf, 2009). As a core molecule in animal immunology and viral disease control, IFN-γ has been shown in numerous mammalian and avian studies to possess broad-spectrum antiviral capacity essential for host antiviral immunity.

In mammals, porcine IFN-γ inhibits the replication of multiple swine viruses, including African swine fever virus (ASFV), vesicular stomatitis virus (VSV), porcine reproductive and respiratory syndrome virus (PRRSV), pseudorabies virus (PRV) and foot-and-mouth disease virus (FMDV) (Rowland et al., 2001; Platanias, 2005; Du et al., 2014; Fan et al., 2020), and its combination with IFN-α produces markedly stronger antiviral effects than either interferon alone.

Avian IFN-γ similarly exhibits stable, broad-spectrum antiviral activity: in chickens, combining IFN-γ with IL-18 or IFN-α markedly enhances inhibition of infectious bursal disease virus (IBDV) (Song et al., 2017). In ducks, recombinant duck IFN-γ suppresses the proliferation of VSV and avian influenza virus (AIV) *in vitro*, and *in vivo* pretreatment reduces the mortality of virus-challenged ducks, alleviates clinical signs, lowers viral shedding, and upregulates interferon-stimulated genes (ISGs) in tissues, confirming the *in vivo* antiviral and immune-activating efficacy of avian IFN-γ (Schultz et al., 2004; Gao et al., 2018).

The antiviral and immunomodulatory functions of IFN-γ depend on the activation of upstream pattern-recognition-receptor signaling. Toll-like receptors (TLRs) are innate immune receptors that recognize viral pathogen-associated molecular patterns and initiate downstream signaling cascades (Brownlie and Allan, 2011; Zhou et al., 2021). Among them, TLR7 specifically senses viral single-stranded RNA (ssRNA) and is a key receptor for host detection of RNA virus infection. Upon binding viral ligands, TLR7 activates the MyD88-dependent pathway, MyD88 mediates the phosphorylation and activation of IRF7, which translocates into the nucleus to drive type I IFN transcription and trigger early antiviral responses (Levy and García-Sastre, 2001; García-Sastre and Biron, 2006). In parallel, TLR7 activation stimulates IFN-γ secretion, and IFN-γ in turn upregulates TLR7 expression, forming a positive feedback loop that amplifies host antiviral immunity (Ramos et al., 2021). As central transcription factors, interferon regulatory factors (IRFs) govern IFN expression and immune activation; IRF7 in particular relays upstream TLR7 signals to downstream IFN secretion (Honda and Taniguchi, 2006). The TLR7–MyD88–IRF7 axis is therefore indispensable for IFN synthesis and antiviral immunity, and the reciprocal TLR7–IFN-γregulation sustains host immune homeostasis. These findings in poultry and mammals provide a theoretical basis for investigating IFN-γ in wild birds.

To date, research on IFN-γ has been largely confined to domestic poultry and terrestrial mammals; the gene structure, biological functions and antiviral regulatory mechanisms of IFN-γ in migratory wild *Larus ridibundus* have not been systematically characterized. Our group has previously cloned several immune genes of *Larus ridibundus*, including TLR7 (GenBank: MZ668652), MyD88 (OR145974), IRF7 (OR145972), IFN-α (OP263971) and Mx (OP263970), providing the genetic resources and preliminary basis for dissecting IFN-γ-mediated immune regulation in this species.

Building on this foundation, the present study cloned the full-length coding sequence of Larus ridibundus IFN-γ and characterized its basic biological properties and *in vitro* antiviral activity. We tested whether the anti-NDV activity of IFN-γ was associated with transcriptional changes of key innate immune genes. By combining gene cloning, recombinant protein production, NDV challenge, and temporal transcriptional analysis in primary lymphocytes, this work provides data on interferon-mediated antiviral responses in a migratory wild bird and a basis for future mechanistic studies.

## 2 Materials and methods

### 2.1 Cloning, bioinformatic analysis of *Larus ridibundus* IFN-γ and preparation of recombinant protein

Peripheral blood collected from 100 *Larus ridibundus* was used as experimental material in this study. Peripheral blood lymphocytes were isolated and purified via density gradient centrifugation. Cells were cultured in complete RPMI 1640 medium supplemented with 12% fetal bovine serum and 1% penicillin-streptomycin mixture at 37 °C with 5% CO_2_, followed by stimulation with concanavalin A (Con A) for cell activation. After cell collection, total RNA was extracted using the Trizol reagent, and the first-strand cDNA was synthesized with a reverse transcription kit.

Specific primers containing *Eco*RIand *Xho*Irestriction enzyme cutting sites were designed based on the published IFN-γ gene sequence of *Larus ridibundus* (**Table 1**). A 498 bp target gene fragment was amplified by PCR. The recovered PCR product was ligated into the pMD18-T vector, and the ligation mixture was transformed into competent *Escherichia coli* DH5α cells. Positive clones were screened by blue-white spot assay, followed by identification via bacterial liquid PCR and double restriction digestion, and verified by sequencing to obtain the correct IFN-γ gene clone. The buffer for double digestion was purchased from Takara Biomedical Technology (Dalian) Co., Ltd., China.

**Table 1:** The primer sequences and fluorescent quantitative primers for the genes.

Gene	Primers	Products(bp)
IFN-γ	F: CGG AAT TCA TG ACT TGC CAG ACC TAC AGC TTC T R: ATC TCG AGT TAG CAT CTG CAC CTC CAC TGA	498
IFN-γ1	F: CAG CTC CAA GAA AGT AAA AGA C R: ACT GAG ACT GGC TCC TTT TC	155
β-Actin	β-Actin F: ACT ACC TCA TGA AGA TCC TGA CA β-Actin R: TCT CCT TCT GCA TCC TGT C	394
NDV NP	NDV NP F: AAC AAA CCA CTC AGG CAA G NDV NP R: CTG TGC TCT CTC TTC AGA CAC	189
TLR7	TLR7 F: ATT TCT GGG ATG TGT GGT AT TLR7 R: TGA ATG CTC TGG GAA AGG T	118
MyD88	MyD88 F: TCG CAA ATT GTA ATG AAC CG MyD88 R: CTG GAA AGT GAT GAG TGT GA	143
IFN-α	IFN-α F: ATC CTC GAC AAC CTC TTC GC IFN-αR: TTT GTT GAT GGT GAG CGT GC	186
Mx	Mx F: TTA TCG CCT ACA AAG AGA GAC ACT CAA Mx R: CTG CCC ACG ACA CTT CAC A	236

Bioinformatic analyses were performed using DNASTAR, MEGA 7.0, and the online tools listed in **Table 2**. NCBI Nucleotide was used for sequence retrieval; ExPASy ProtParam and ProtScale were used to examine physicochemical properties and hydrophobicity; SignalP 6.0 and DeepTMHMM were used to predict signal peptide and transmembrane regions; Euk-mPLoc 2.0 was used for subcellular localization; NetNGlyc 1.0 and NetPhos 3.1 were used to predict glycosylation and phosphorylation sites; SOPMA and SWISS-MODEL were used for secondary- and tertiary-structure prediction; and MEME-MAST and MEGA 7.0 were used for motif and phylogenetic analyses. The target gene was inserted into pET32a and transformed into Transetta (DE3) cells. Induction temperature, induction duration, and IPTG concentration were optimized by gradient screening. Following large-scale induction, cell pellets were disrupted by low-temperature sonication, inclusion bodies were washed with a urea gradient, the recombinant protein was refolded by gradient dialysis, and protein concentration was determined by the Bradford method.

**Table 2:** Bioinformatics analysis software and functions.

Software name & website	Function
NCBI Nucleotide (https://www.ncbi.nlm.nih.gov/nucleotide)	Sequence retrieval
Expasy ProtParam tool (https://web.expasy.org/protparam/)	Physicochemical property analysis of protein
SignalP 6.0 (http://www.cbs.dtu.dk/services/)	Protein signal peptide analysis
DeepTMHMM (http://www.cbs.dtu.dk/services/)	Protein transmembrane domain analysis
Euk-mPLoc 2.0 (http://www.csbio.sjtu.edu.cn/bioinf/Cell-PLoc-2/)	Protein subcellular localization analysis
NetNGlyc-1.0 (http://www.cbs.dtu.dk/services/)	Protein glycosylation site analysis
NetPhos-3.1 (http://www.cbs.dtu.dk/services/)	Protein phosphorylation site analysis
SOPMA (https://prabi.ibcp.fr/htm/site/web/app.php/home)	Protein secondary structure analysis
SWISS MODEL (https://swissmodel.expasy.org/)	Protein tertiary structure prediction
Antigenic Peptides (http://imed.med.ucm.es/Tools/antigenic.pl)	Antigenic determinant analysis
Expasy ProtScale (https://web.expasy.org/protscale/)	Protein hydrophobicity analysis
MEME-MAST (https://meme-suite.org/meme/doc/meme.html)	Protein conserved motif analysis

The base refolding buffer contained 50 mM Tris-HCl (pH 8.0), 150 mM NaCl, 1 mM EDTA, 2 mM reduced glutathione, and 0.5 M L-arginine. Serial urea-gradient buffers were prepared with this base buffer, and recombinant IFN-γ was refolded by gradient dialysis. The refolded protein was used to immunize New Zealand White rabbits for production of polyclonal antiserum. Antibody titers were evaluated by double agar diffusion and indirect ELISA. Immunoglobulins were purified by saturated ammonium sulfate-sodium octanoate precipitation, and Western blotting was used to assess antibody specificity and the immunoreactivity of the recombinant protein.

### 2.2 Detection of viral load and innate immune gene transcription in NDV-infected *Larus ridibundus* lymphocytes

#### 2.2.1 NDV viral titration

Viral titers were quantified by the 50% tissue culture infectious dose (TCID_50_) assay. DF-1 cells were seeded in 96-well plates, and virus-containing suspensions were serially diluted 10-fold. Each dilution (100 µL/well) was incubated with cells for 1 h at 37 °C. Unbound virions were removed by PBS washing, and maintenance medium (DMEM containing 2% FBS) was added. Cytopathic effects were recorded during 3–5 days after infection, and TCID_50_ values were calculated using the Reed-Muench method.

#### 2.2.2 Primer design for qRT-PCR

Specific qPCR primers for NDV NP, β-Actin, IFN-γ, TLR7, MyD88, IFN-α, and Mx were designed using Primer Premier 6.0 from the reference sequences listed in **Table 1**. Primer sequences and expected amplicon sizes are provided in **Table 1**, including the 189-bp NDV NP (GenBank No. JX524203) and 394-bp β-Actin products (GenBank No. NM001303173.1). qRT-PCR was performed in a 20-µL reaction using the reagent composition and thermal cycling program shown in **Table 3**.

**Table 3:** Fluorescent quantitative PCR reaction system and fluorescent quantitative primers for the genes.

Reagent	Volume (μL)	Temperature	Time
2×SuperReal PreMix Plus(SYBR Green)	10	95°C	15 min
Forward primer	1	95°C	10 s
Reverse primer	1	50°C	20 s 39
Standard plasmid template	1	72°C	30 s
ddH_2_O	7		
Total volume	20		

Each qRT-PCR reaction was performed in a total volume of 20 μL, consisting of 10 μL of 2×SuperReal PreMix Plus (SYBR Green), 1 μL of forward primer, 1 μL of reverse primer, 1 μL of standard plasmid template, and 7 μL of ddH_2_O. The thermocycling program was set as follows: an initial pre-denaturation step at 95 °C for 15 min, followed by 39 cycles of 95 °C for 10 s, 50 °C for 20 s, and 72 °C for 30 s. Melting-curve analysis was routinely performed after amplification to verify the specificity of PCR products, and no-template controls were included for each primer pair to exclude contamination. Primer sequences and expected amplicon sizes for the detected genes, including NDV NP, β-actin, IFN-γ, TLR7, MyD88, IRF7, IFN-α, and Mx, are listed in **Table 1**. The β-actin gene was used as an internal reference for normalization. All experimental treatments were performed with three independent biological replicates, and the relative transcript levels of target genes were calculated as described in Section 2.5. These details are reported to improve assay transparency in line with MIQE principles (Bustin et al., 2009).

#### 2.2.3 Gradient NDV infection for optimal dose screening

Purified peripheral blood lymphocytes of L. ridibundus were adjusted to 1.0 × 10^6^ cells/mL for culture. Three infection groups (low, medium, high viral dose) were established to compare viral replication levels under different viral loads. Each group received 250 μL, 500 μL and 750 μL calibrated NDV stock, respectively, with culture medium supplemented to equal volume. Cells were incubated at 37 °C, 5% CO_2_ for 24 h, 36 h, 48 h and 60 h. Cells were collected at each time point for total RNA extraction and reverse transcription into cDNA. qRT-PCR was used to detect NP transcriptional abundance, and the optimal infection dose with moderate viral replication and intact cell status was selected for subsequent temporal experiments.

To further verify viral protein expression at translational level, cell samples collected at 24 h and 60 h post infection were subjected to Western blot analysis of NP protein. Harvested cells were lysed in 2× protein loading buffer (20-mM Tris-HCl, 2% SDS, 100-mM dithiothreitol, 20% glycerol, and 0.016% bromophenol blue), denatured, and resolved by 10% SDS-PAGE. Proteins were transferred to nitrocellulose membranes. Each membrane was blocked in skim milk for 2 h at room temperature, incubated with primary antibodies overnight at 4 °C, then washed three times with Tris-buffered saline with Tween 20 (TBS-T) [50-mM Tris-HCl (pH 7.6), 150-mM NaCl, and 0.1% Tween 20] for 10 min. Next, membranes were incubated with secondary antibodies for 2 h at room temperature and washed three times with TBS-T for 10 min. Protein bands were visualized using the Chemiluminescent Imaging System (Bio Tanon, Shanghai, China).

#### 2.2.4 Dynamic detection of host innate immune genes

Using the selected NDV dose, lymphocytes were infected and harvested at 12, 24, 36, 48, and 60 h post-infection to quantify TLR7, MyD88, IRF7, IFN-α, IFN-γ, and Mx transcripts with the primers listed in **Table 1**. Three independent biological replicates were included for each treatment and time point. Uninfected lymphocytes served as the blank negative control, and all Ct values were retained for relative-expression analysis.

### 2.3 Detection of transcription levels of related genes in lymphocytes incubated with recombinant IFN-γ protein

Lymphocytes were assigned to low-dose (50 ng/mL), medium-dose (200 ng/mL), and high-dose (350 ng/mL) recombinant IFN-γ groups and cultured in 6-well plates. Cells were collected after 12, 24, 36, 48, and 60 h. At each time point, total RNA was extracted and reverse transcribed, and TLR7, MyD88, IRF7, IFN-α, IFN-γ, and Mx transcripts were quantified using the qRT-PCR conditions in **Table 3**. Each condition included three independent biological replicates. The concentration producing the strongest and most stable transcriptional response was selected for the subsequent antiviral assay.

### 2.4 Detection of transcription levels of related genes after combined intervention with recombinant IFN-γ protein and NDV

Four conditions were analyzed: an untreated blank control, an NDV-only infection control, an IFN-γ-only treatment control (200 ng/mL), and a combined IFN-γ + NDV group. For the combined group, lymphocytes were exposed to 200 ng/mL purified refolded recombinant IFN-γ for 10 h before addition of the selected medium NDV dose and were then co-incubated. Cell samples were collected at 12, 24, 36, 48, and 60 h. Total RNA was extracted and reverse transcribed, and NDV NP, TLR7, MyD88, IRF7, IFN-α, IFN-γ, and Mx transcripts were quantified by qRT-PCR. Each condition included three independent biological replicates, and all Ct values were retained for statistical analysis.

### 2.5 Statistical analysis of data

Raw qRT-PCR Ct values were organized in Microsoft Excel, and relative transcription was calculated using the Pfaffl method (Pfaffl, 2001). Statistical analyses and graphs were generated in GraphPad Prism 9.5. Data are presented as mean ± SEM from three independent biological replicates. Differences among treatment groups or time points were evaluated by one-way ANOVA. *P < 0.05* was considered statistically significant, *P < 0.01* was considered highly significant, and *P > 0.05* was considered not significant.

## 3 Results

### 3.1 Cloning and pET32a-IFN-γ recombinant expression plasmid construction of the CDS region of *Larus ridibundus* IFN-γ gene

**Figure 1:**
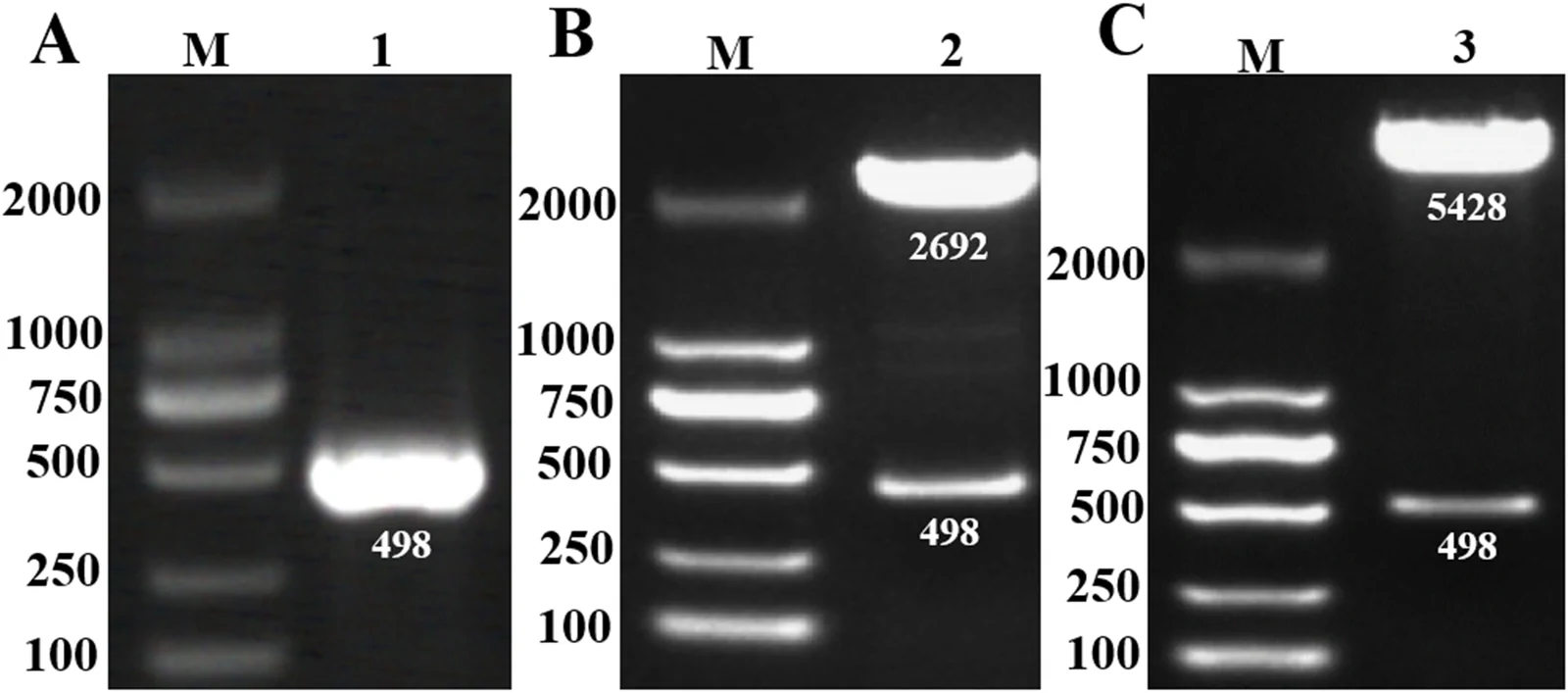
PCR and double restriction digestion identification of recombinant plasmid pMD18-T-IFN-γ and recombinant prokaryotic expression vector pET32a-IFN-γ. **(A)** PCR amplification of the IFN-γ gene of the Larus ridibundus. M, DL2000 DNA Maker; 1. CDS region PCR amplification of the IFN-γ gene; **(B)** double digestion validation of recombinant plasmid pMD18-T-IFN-γ. M, DL2000 DNA Maker; 2. double digestion verification of pMD18-T-IFN-γ; **(C)** double digestion validation of the recombinant prokaryotic expression plasmid pET32a-IFN-γ. M, DL2000 DNA Maker; 3. double digestion validation of the recombinant prokaryotic expression plasmid pET32a-IFN-γ.

PCR amplification generated a single 498-bp IFN-γ coding-sequence band. PCR analysis of pMD18-T recombinants produced the expected 498-bp product, and double digestion yielded 2692-bp vector and 498-bp insert bands (**Figures 1A, B**). The sequence matched the expected target and was deposited in GenBank under accession MZ713251. Double digestion of pET32a-IFN-γ yielded the expected 5900-bp vector and 498-bp insert bands (**Figure 1C**), and sequencing confirmed successful construction of the expression plasmid.

### 3.2 Bioinformatic analysis of *Larus ridibundus* IFN-γ protein

The physicochemical properties of *Larus ridibundus* IFN-γ protein were predicted via ExPASy ProParam tool. This protein is encoded by 165 amino acids with a molecular weight of 19.16 kDa and a total atomic count of 2698. Serine, leucine, and lysine ranked as the top three amino acids in abundance, while proline, tyrosine, and cysteine were the least abundant. The proportions of basic and acidic amino acids were 17.58% and 11.52%, respectively. The theoretical isoelectric point (pI) of the protein was 8.97. A total of 13 rare codons were identified in the gene sequence, including five AGG, two AGA, three GGA and three AUA, with no consecutive rare codons detected.

**Figure 2:**
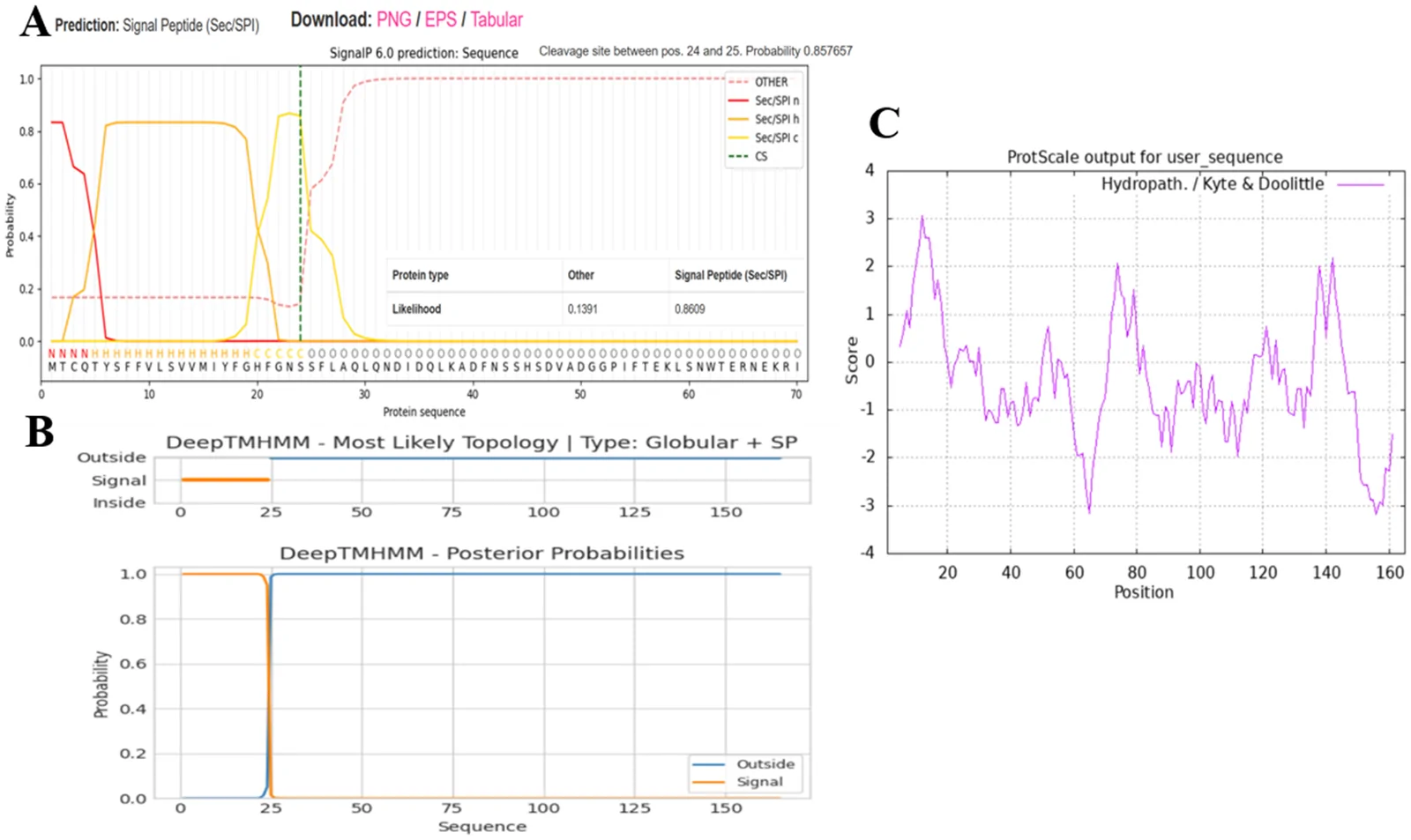
Signal peptide cleavage site, transmembrane region and hydrophobicity analysis of Larus ridibundus IFN-γ. **(A)** Results of signaling peptide cleavage site analysis in Larus ridibundus IFN-γ; **(B)** Results of transmembrane region analysis in Larus ridibundus IFN-γ; **(C)** Results of hydrophobicity analysis in Larus ridibundus IFN-γ.

Conserved domain analysis demonstrated that this protein belongs to the IFN-γ superfamily. Signal peptide and transmembrane region prediction revealed that the signal peptide cleavage site was located between amino acids 24 and 25 (**Figure 2A**). DeepTMHMM topological prediction revealed that residues 1–25 of IFN-γ correspond to a canonical signal peptide (**Figure 2B**), and the remaining polypeptide forms an extracellular soluble globular domain with no transmembrane helices, consistent with signal peptide and subcellular localization prediction outcomes. Hydrophobicity analysis revealed the presence of three hydrophobic regions within the protein, with an overall GRAVY (grand average of hydropathicity) score of −0.39 (**Figure 2C**). Hydrophilic amino acids accounted for 62.42% and hydrophobic amino acids accounted for 37.58% of the total residues.

**Figure 3:**
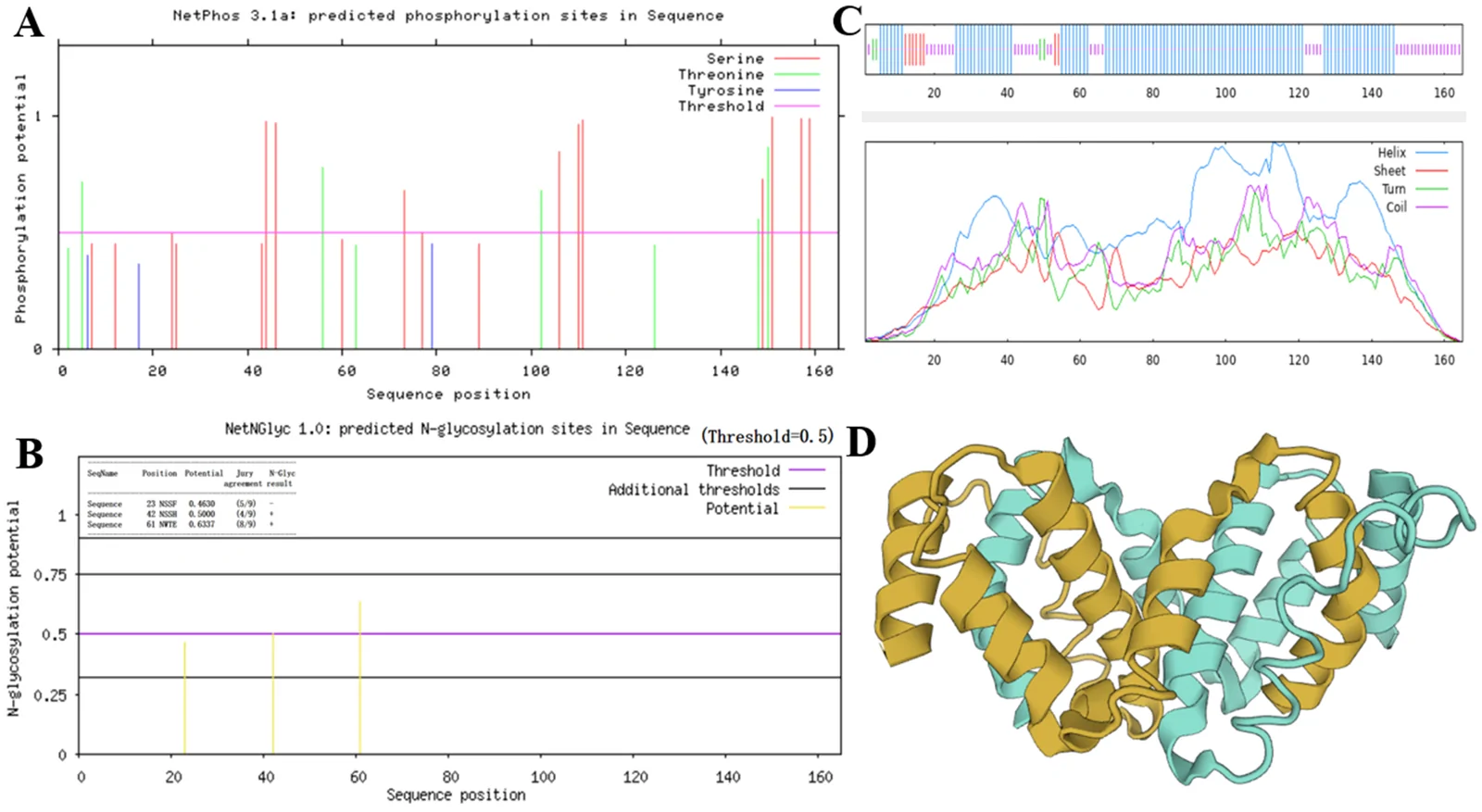
Prediction of phosphorylation sites, N-glycosylation sites, secondary structure and tertiary structure of Larus ridibundus IFN-γ. **(A)** Results of phosphorylation site analysis in Larus ridibundus IFN-γ; **(B)** Results of N-terminal glycosylation site analysis in Larus ridibundus IFN-γ; **(C)** Results of secondary structure analysis in Larus ridibundus IFN-γ; **(D)**. Results of tertiary structural analysis in Larus ridibundus IFN-γ.

Antigenicity analysis identified six antigenic determinants, and the average antigenic propensity of the whole protein was 1.0264. Post-translational modification site prediction identified 3 palmitoylation sites (**Figure 3A**), 2 N-glycosylation sites (**Figure 3B**) and 15 phosphorylation sites (6 serine phosphorylation sites and 9 threonine phosphorylation sites, without tyrosine phosphorylation sites). Secondary structure prediction showed that α-helix was the dominant structural element (64.85%), followed by random coil (27.88%), while extended strand and β-turn occupied relatively low proportions (**Figure 3C**). Homology modeling of the tertiary structure was performed based on the crystal structures of human and bovine IFN-γ, with a sequence identity of 40.17% and a confidence score of 0.55. The protein exists as a homodimer (**Figure 3D**).

**Figure 4:**
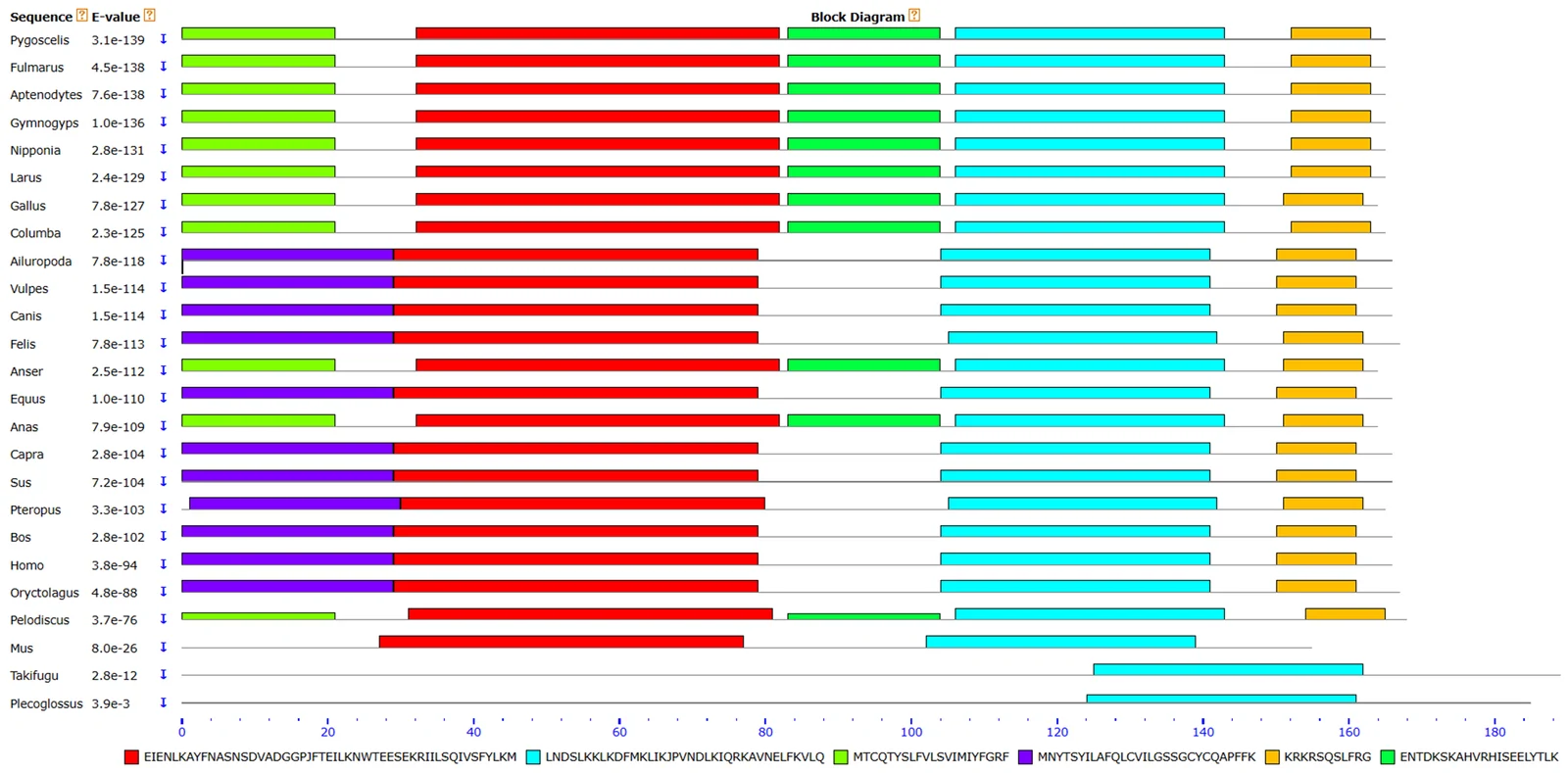
Results of conserved region analysis in Larus ridibundus IFN-γ protein sequence.

**Figure 5:**
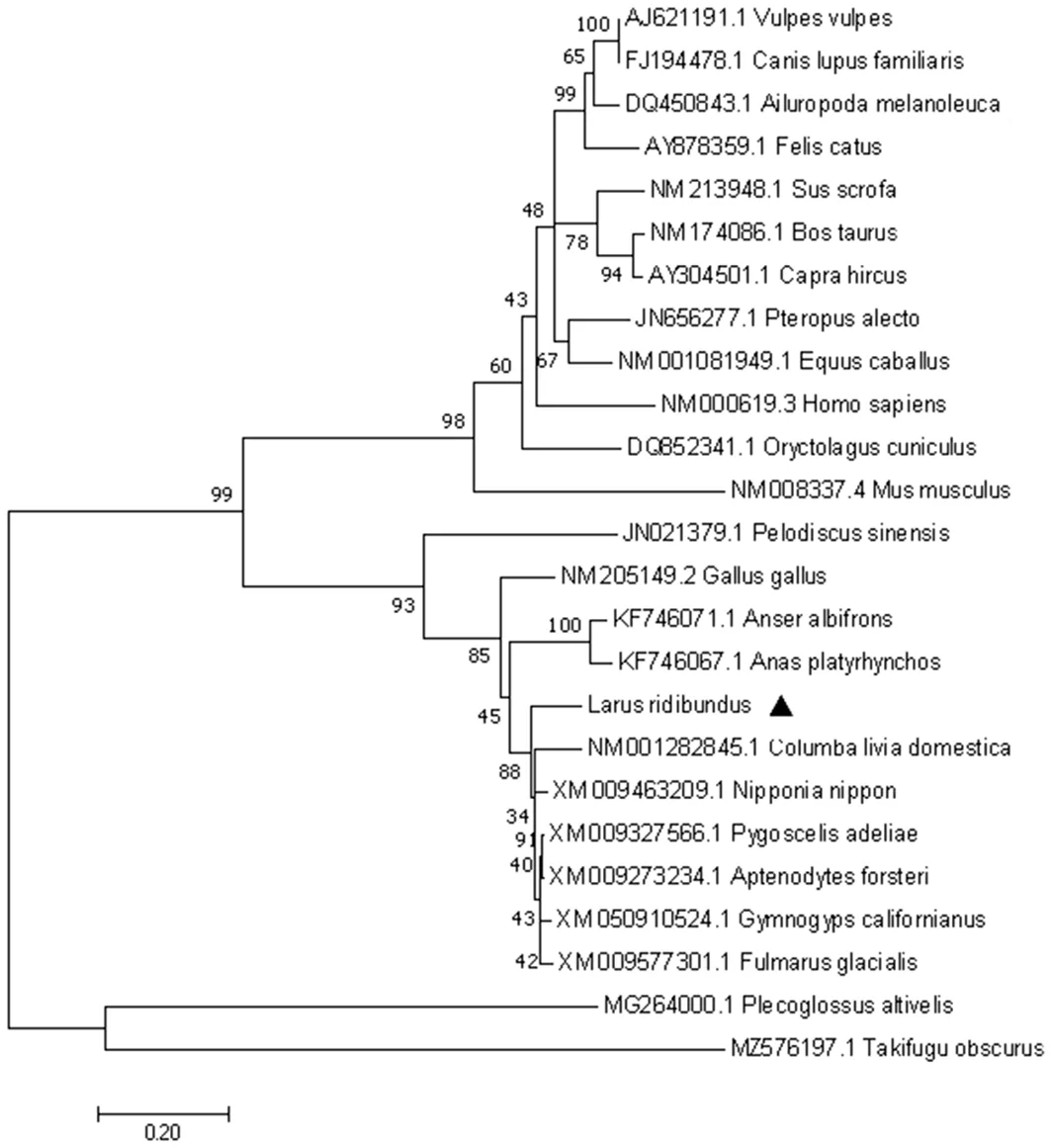
Results of evolutionary tree construction in Larus ridibundus IFN-γ CDS.

Homology alignment results showed that the sequence identity of *Larus ridibundus* IFN-γ CDS with homologous sequences from 24 species ranged from 36.6% to 92.2%. The highest homology was observed with Adélie penguin IFN-γ, and the lowest with ayu. Multiple conserved motif analysis identified 5 conserved motifs shared between this protein and avian homologous sequences (**Figure 4**), 4 motifs shared with mammals, and only 1 motif shared with fish. Phylogenetic tree analysis indicated that *Larus ridibundus* IFN-γ had the closest genetic relationship with avian species (rock pigeon, crested ibis, mallard), whereas the evolutionary distance from mammals and fish was relatively far, conforming to the general rules of species evolution (**Figure 5**).

### 3.3 Preparation of recombinant *Larus ridibundus* IFN-γ protein

**Figure 6:**
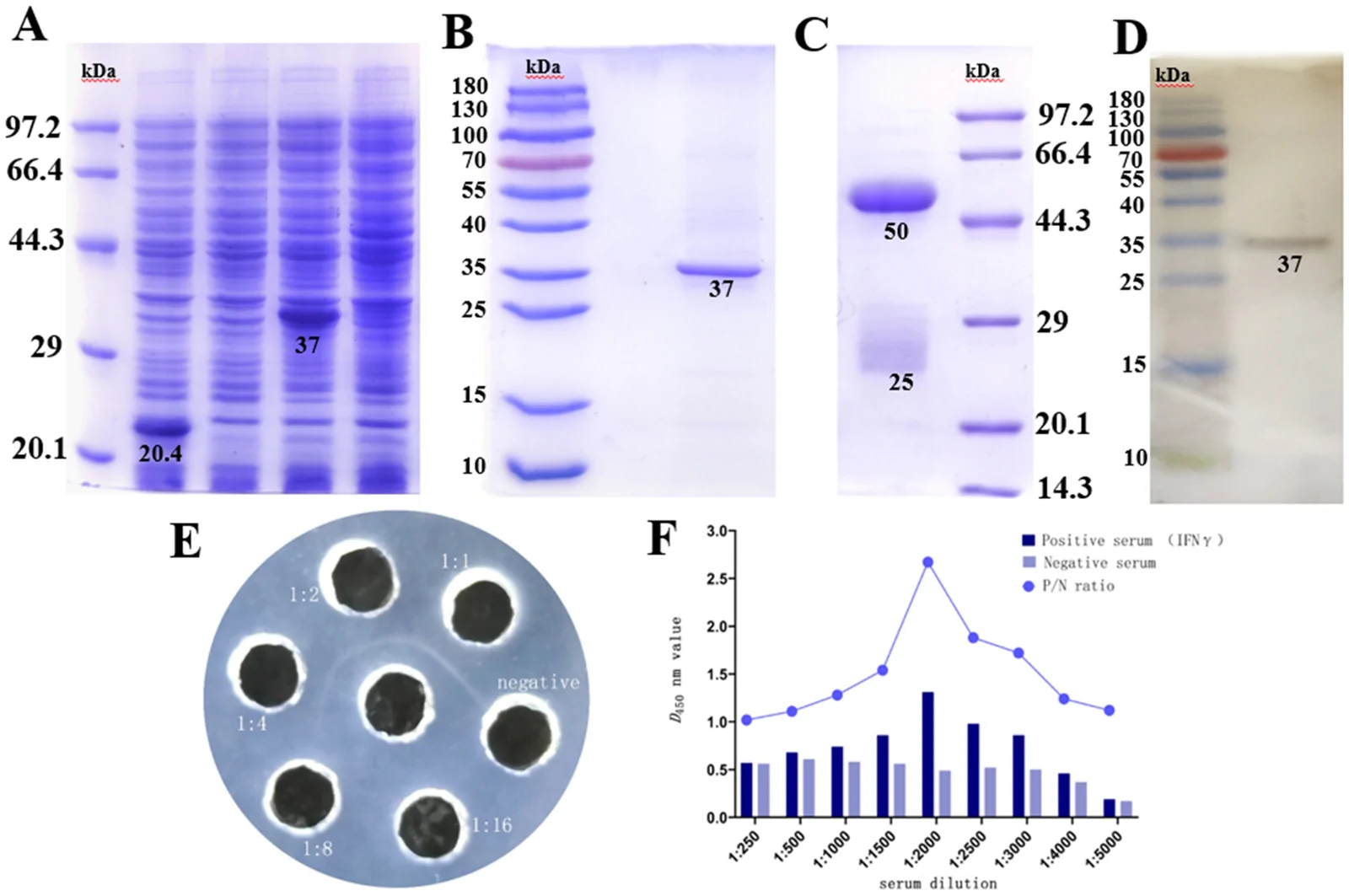
Analysis of recombinant protein induced expression and purification, crude purity assessment, as well as serum IgG titer measurement by bidirectional agar diffusion and indirect ELISA. **(A)** Induced expression results of recombinant protein IFN-γ; **(B)** Results of purification analysis of recombinant proteins; **(C)** Analysis of seru IgG crude purity results; **(D)** Western blot analysis of recombinant protein IFN-γ; **(E)** Determination of serum titer by bidirectional agar diffusion method; **(F)** Indirect ELISA method to determine serum titer results.

Following IPTG induction of Transetta (DE3) cells carrying pET32a-IFN-γ, SDS-PAGE revealed a specific band at approximately 37 kDa, consistent with the expected size of the pET32a-IFN-γ fusion protein rather than mature IFN-γ alone. The empty pET32a control produced a band near 20 kDa, whereas the uninduced group showed no corresponding specific band (**Figure 6A**). Optimal induction was achieved at 37 °C for 5 h with 0.6 mmol/L IPTG. The recombinant protein was predominantly present in inclusion bodies and was enriched by inclusion-body washing and Ni-affinity purification before gradient-dialysis refolding (**Figure 6B**). The Bradford standard curve was y = 0.001359x + 0.07306 (R^2^ = 0.9925), and the final protein concentration was 384.21 µg/mL.

Purified recombinant protein was used to immunize New Zealand white rabbits, and serum was separated from collected blood samples. The antibody titer was 1:4 by double agar gel diffusion (**Figure 6E**). Indirect ELISA showed a serum antibody titer of 1:2000 when the antigen coating amount was 20 µg (**Figure 6F**). This result indicated that the immunized rabbits produced a detectable antibody response to the recombinant antigen. Rabbit-derived polyclonal IgG was purified by saturated ammonium sulfate-sodium octanoate precipitation. SDS-PAGE showed 50-kDa heavy-chain and 25-kDa light-chain bands, consistent with IgG enrichment (**Figure 6C**). Western blotting detected a specific band at approximately 37 kDa. This result supported specific recognition of the pET32a-IFN-γ fusion protein by the prepared polyclonal antibody (**Figure 6D**).

### 3.4 Transcriptional changes of TLR7 pathway-related genes in NDV-infected lymphocytes

**Figure 7:**
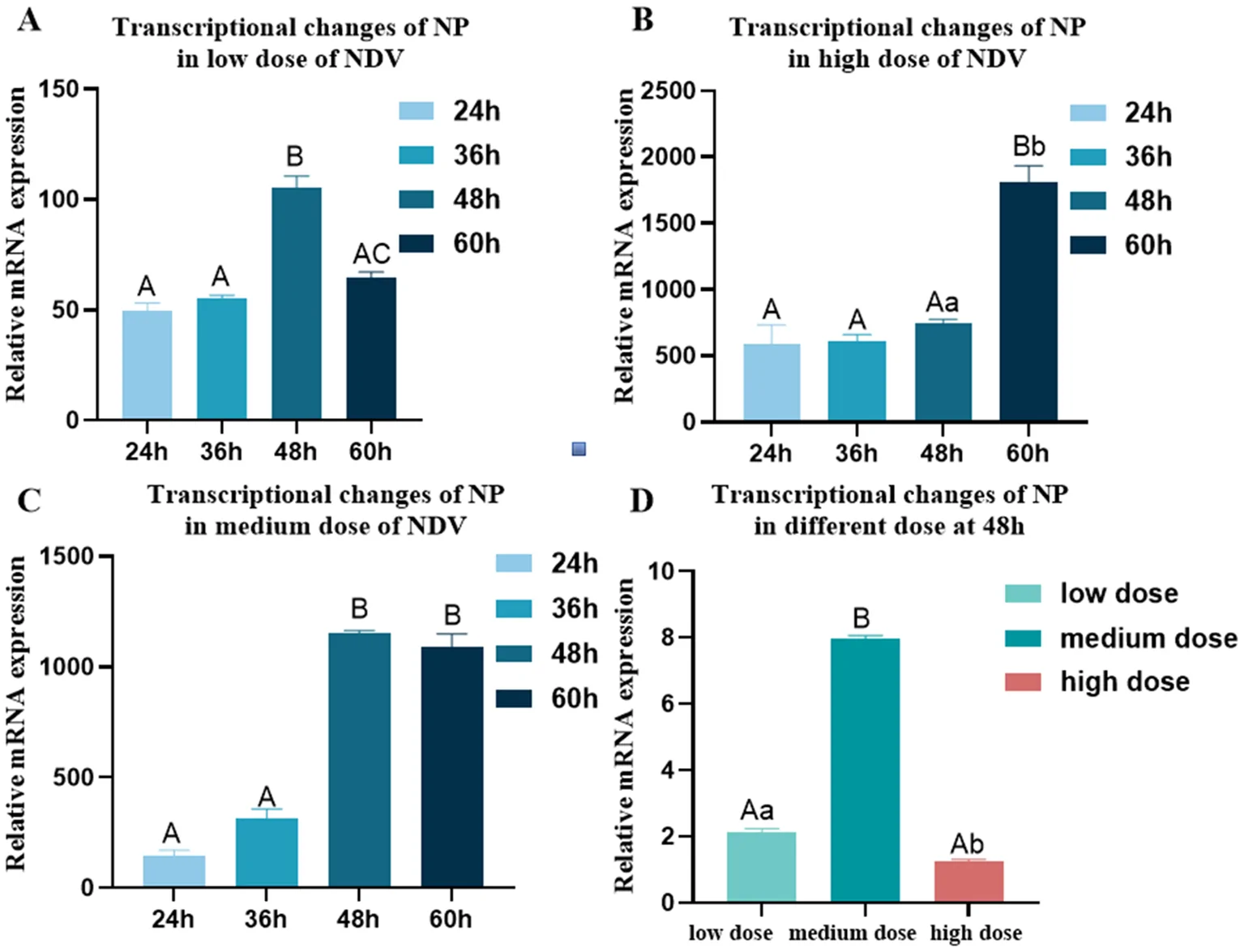
Transcriptional changes of the NP gene in lymphocytes following infection with different doses of NDV. **(A)** NP transcriptional dynamics under low-dose NDV infection; **(B)** NP transcriptional dynamics under medium-dose NDV infection; **(C)** NP transcriptional dynamics under high-dose NDV infection; **(D)** Comparison of NP transcript levels among different infection doses at 48 h. The same uppercase letters indicate no significant difference (*P*>0.05), whereas different uppercase letters indicate extremely significant differences (*P* < 0.01). Different lowercase letters indicate significant differences (*0.05<P<0.01*), whereas the same lowercase letters indicate no significant differences (*P>0.05).*.

**Figure 8:**
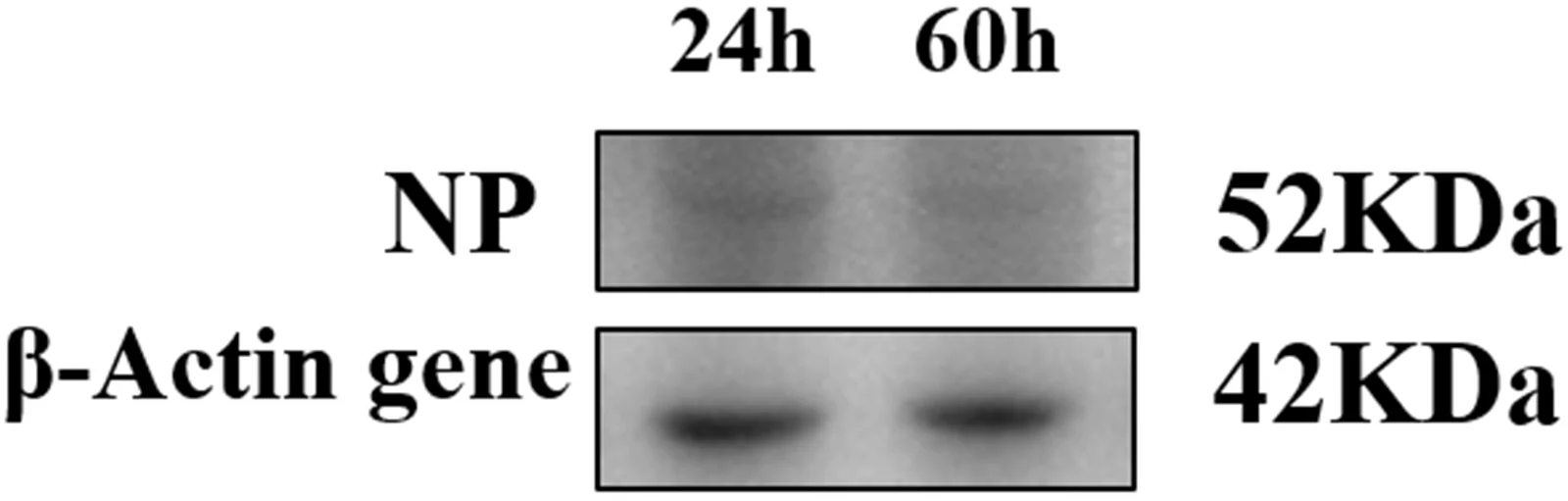
The expression of NDV NP protein at 24 h and 60 h was detected via Western blot at 60h in medium dose. NP: NDV NP protein; β-Actin gene.

**Figure 9:**
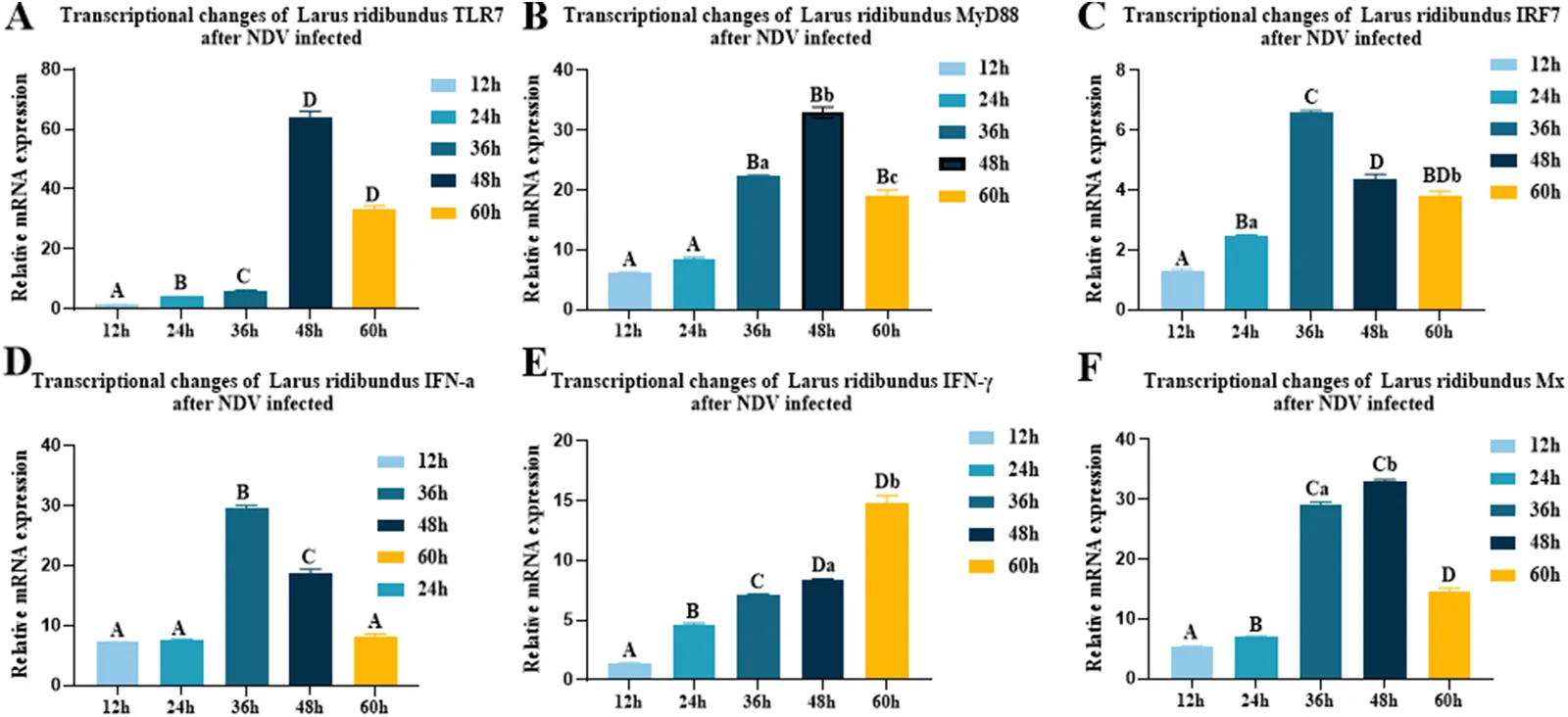
Transcriptional changes of pathway-related genes in lymphocytes infected with medium-dose NDV. **(A)** Transcriptional changes of Larus ridibundus TLR7 after NDV infected; **(B)** Transcriptional changes of Larus ridibundus MyD88 after NDV infected; **(C)** Transcriptional changes of Larus ridibundus IRF7 after NDV infected; **(D)** Transcriptional changes of Larus ridibundus IFN-a after NDV infected; **(E)**. Transcriptional changes of Larus ridibundus IFN-γ after NDV infected; **(F)**. Transcriptional changes of Larus ridibundus Mx after NDV infected. The same uppercase letters indicate no significant difference (*P*>0.05), whereas different uppercase letters indicate extremely significant differences (*P* < 0.01). Different lowercase letters indicate significant differences (*0.05<P<0.01*), whereas the same lowercase letters indicate no significant differences (*P>0.05).*.

The TCID_50_ of NDV was calculated as 10^–3.5^ per 0.1 mL using the Reed-Muench formula. Cells were incubated with equal culture medium supplemented with 250 μL, 500 μL and 750 μL of the above NDV stock solution, corresponding to total viral doses of 2.5×10^–2.5^, 5×10^–2.5^ and 7.5×10^–2.5^ TCID_50_ per well, respectively. Low-dose NDV leads to weak viral replication in lymphocytes, while high-dose NDV causes rapid cell death and unstable NP gene expression. Medium-dose NDV induces stable NP transcription: NP mRNA levels gradually rise starting at 24 h, peak significantly at 48 h, and slightly decline at 60 h with no significant difference compared with the 48 h group (**Figures 7A–D**). Therefore, medium-dose NDV was selected for subsequent detection. NP protein showed clear expression at 24 h and 60 h under this treatment condition (**Figure 8**). Lymphocytes were stimulated with medium-dose NDV (5×10^–2.5^ TCID_50_), and cell samples were harvested at 12 h, 24 h, 36 h, 48 h and 60h post-stimulation for quantitative detection of transcriptional dynamics of TLR7, MyD88, IRF7, IFN-α, IFN-γ and Mx genes. The results were as follows: The transcription abundance of TLR7 peaked at 48 h, with extremely significant differences in transcription levels among time points (*P<0.01*) (**Figure 9A**). MyD88 transcription also reached its peak at 48 h, with a declining trend at earlier time points; significant differences were observed between 48 h and 60 h (*0.01<P<0.05*) (**Figure 9B**).

IRF7 transcription peaked at 36 h with overall extremely significant expression differences across time points (*P<0.01*), followed by a decline from 48 h to 60 h, with no significant difference between 48 h and 60 h (*P>0.05*) (**Figure 9C**). The transcription peak of IFN-α appeared at 36 h, and its expression was markedly downregulated from 48 h to 60 h, with extremely significant transcriptional differences (*P<0.01*) (**Figure 9D**). The transcription level of IFN-γ continuously increased from 12 h to 60 h with prolonged stimulation, showing extremely significant differences among different time points (*P<0.01*) (**Figure 9E**). Mx transcription significantly increased starting at 36 h and reached the maximum at 48 h; the peak value showed significant differences compared with other time points (*0.01<P<0.05*). Mx expression dropped sharply at 60 h, with an extremely significant difference between 48 h and 60 h (*P<0.01*) (**Figure 9F**).

### 3.5 Transcriptional changes of TLR7 pathway genes in lymphocytes treated with different doses of IFN-γ protein

**Figure 10:**
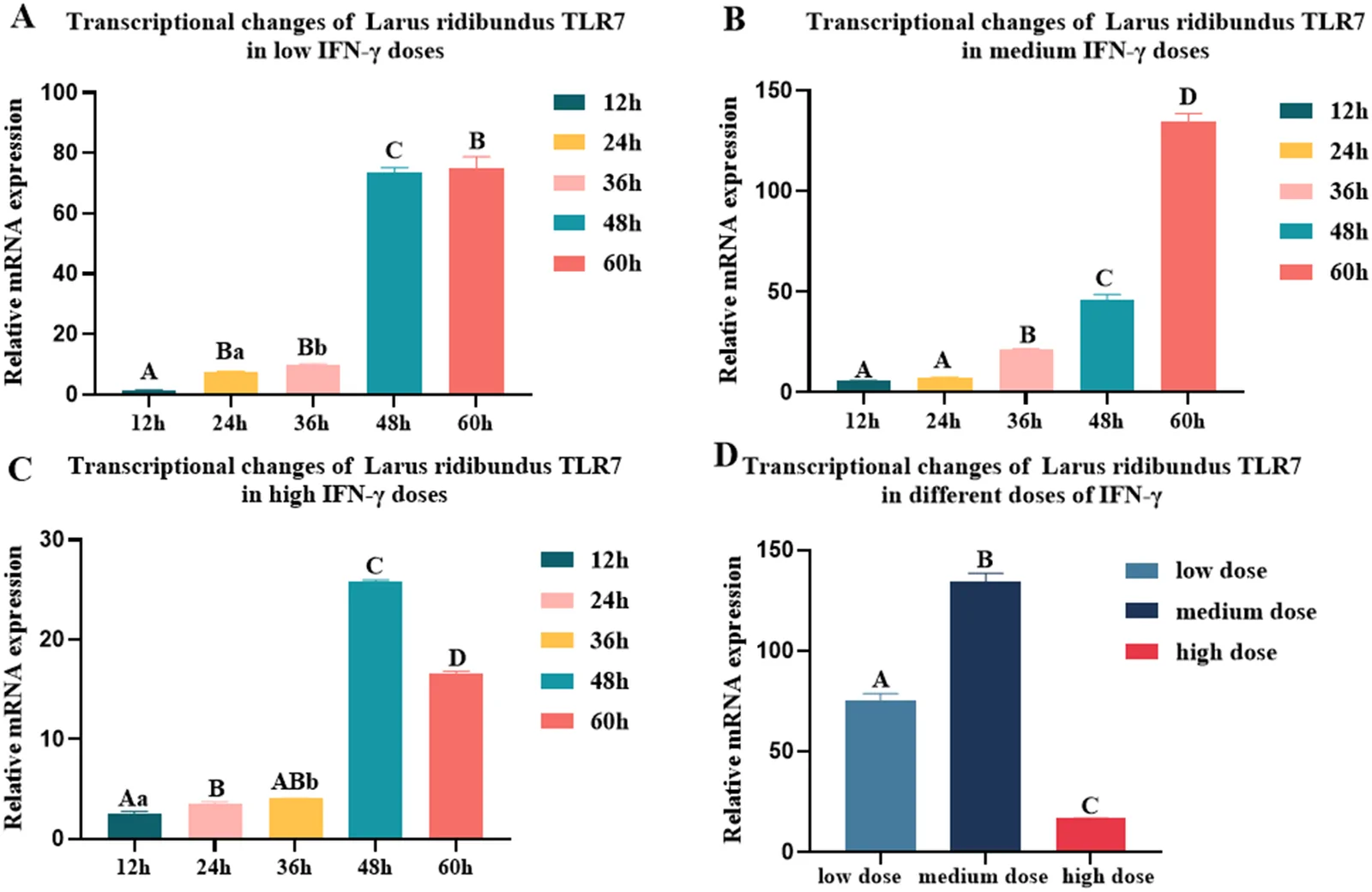
Transcriptional changes of TLR7 in lymphocytes stimulated with different doses of IFN-γ. **(A)** Transcriptional changes of Larus ridibundus TLR7 in low IFN-γ doses; **(B)** Transcriptional changes of Larus ridibundus TLR7 in medium IFN-γ doses; **(C)** Transcriptional changes of Larus ridibundus TLR7 in high IFN-γ doses; **(D)** Comparison of transcriptional changes of Larus ridibundus TLR7 in different IFN-γ doses at 48h. The same uppercase letters indicate no significant difference (*P>0.05*), whereas different uppercase letters indicate extremely significant differences (*P<0.01*). Different lowercase letters indicate significant differences (*0.05<P<0.01*), whereas the same lowercase letters indicate no significant differences (*P>0.05*).

Previous transcriptional detection of virus-infected models showed that IFN-γ maintained persistently high transcription levels. To clarify the regulatory effects of IFN-γ at different concentrations on key genes of the TLR7 signaling pathway and screen the optimal functional dose of IFN-γ, lymphocytes were stimulated *in vitro* with serially diluted IFN-γ protein. Cell samples were collected at 12 h, 24 h, 36 h, 48 h and 60 h post-stimulation, and dynamic transcriptional changes of the TLR7 gene were quantified by qRT-PCR. The gradient stimulation results were summarized as follows:

In the low-dose IFN-γ group, TLR7 transcription increased over time and peaked at 60 h; the difference between 48 and 60 h was highly significant (*P < 0.01*; **Figure 10A**). Medium-dose IFN-γ induced a higher peak TLR7 transcript level than the low dose (*P < 0.01*; **Figure 10B**). High-dose IFN-γ produced a biphasic response, with TLR7 transcription increasing and then declining; the level at 60 h was significantly lower than that at 48 h (*P < 0.01*; **Figure 10C**). At 48 h, the medium-dose group showed the strongest TLR7 transcriptional response among the three concentrations (*P < 0.01*; **Figure 10D**).

### 3.6 Transcriptional changes of other pathway genes in lymphocytes treated with medium-dose IFN-γ protein

**Figure 11:**
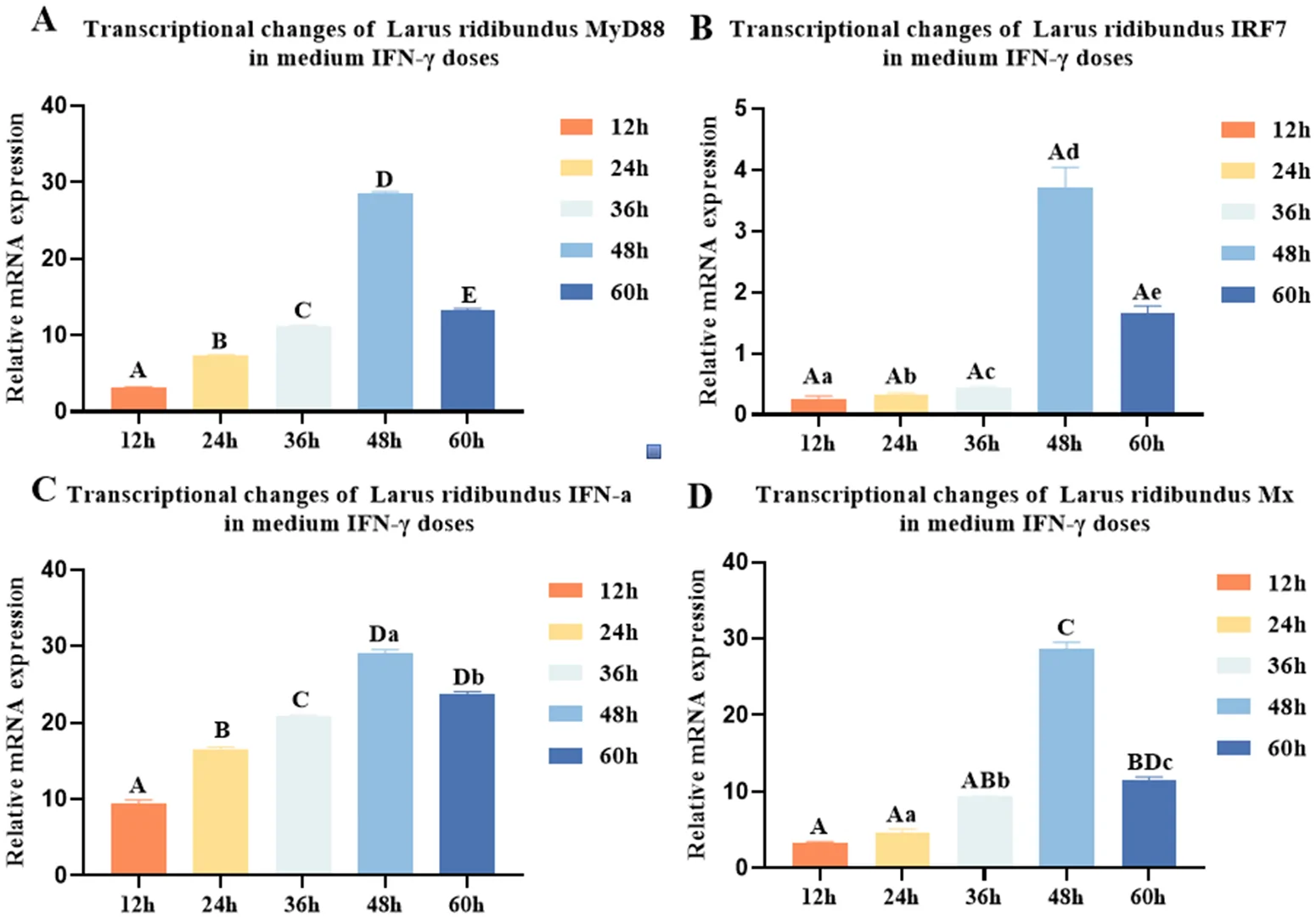
Transcriptional alterations of other pathway-related genes in lymphocytes stimulated with medium doses of IFN-γ. **(A)** Transcriptional changes of Larus ridibundus MyD88 in medium doses; **(B)** Transcriptional changes of Larus ridibundus IRF7 in medium doses; **(C)** Transcriptional changes of Larus ridibundus IFN-a in medium doses; **(D)** Transcriptional changes of Larus ridibundus Mx in medium doses. The same uppercase letters indicate no significant difference (*P>0.05*), whereas different uppercase letters indicate extremely significant differences (*P<0.01*). Different lowercase letters indicate significant differences (*0.05<P<0.01*), whereas the same lowercase letters indicate no significant differences (*P>0.05).*.

Following stimulation of *Larus ridibundus* lymphocytes with 200 ng/mL IFN-γ, MyD88, IRF7, IFN-α, and Mx transcripts increased from 12 to 48 h and reached their maximum levels at 48 h before declining at 60 h. IRF7 followed this time-dependent pattern and differed significantly among time points (*0.01 < P < 0.05*; **Figure 11B**). MyD88 and IFN-α showed highly significant differences among time points (*P < 0.01*; **Figures 11A, C**), and Mx showed highly significant differences during 36–60 h (*P < 0.01*; **Figure 11D**).

### 3.7 Transcriptional changes of pathway genes in lymphocytes co-treated with medium-dose NDV and medium-dose IFN-γ protein

**Figure 12:**
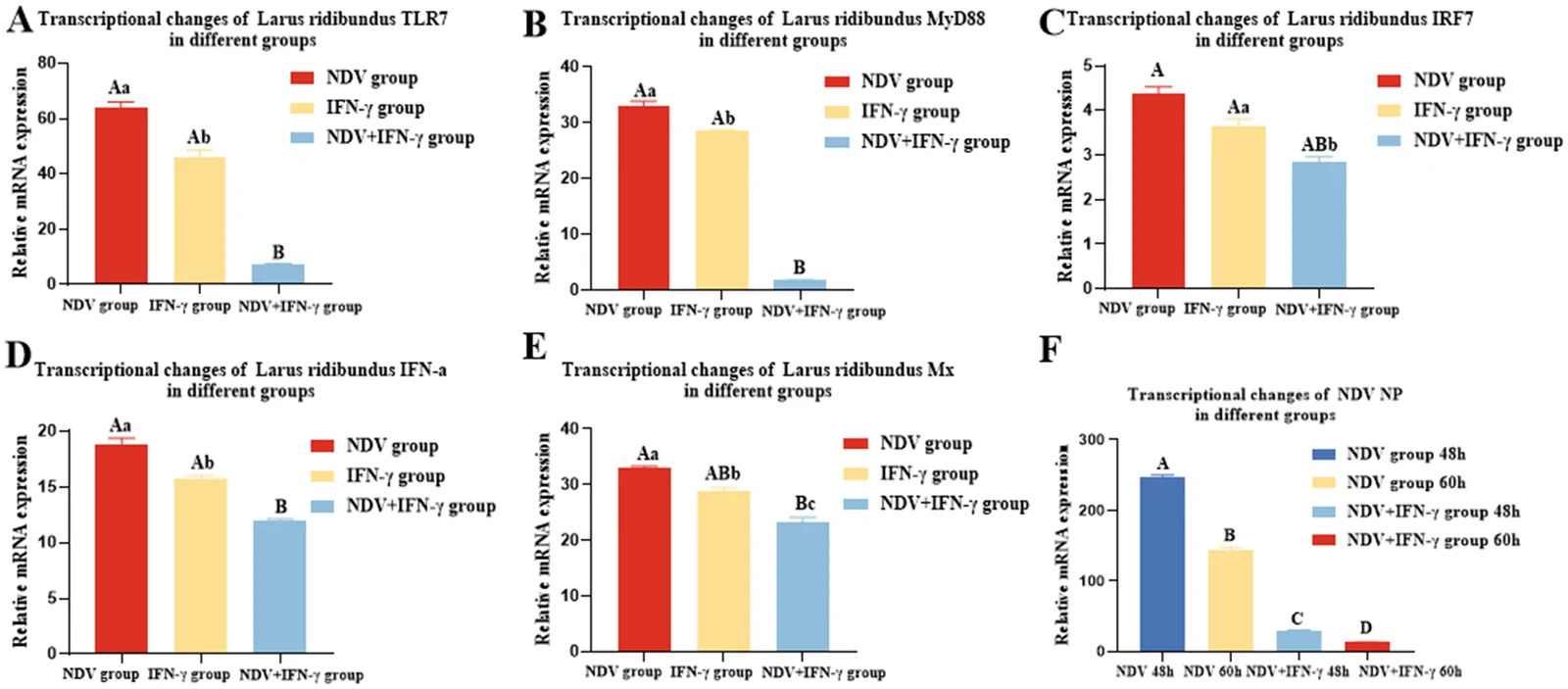
Transcriptional alterations of NP and pathway-related genes in NDV-infected lymphocytes treated with IFN-γ. **(A)** Transcriptional changes of Larus ridibundus TLR7 in different groups; **(B)** Transcriptional changes of Larus ridibundus MyD88 in different groups; **(C)** Transcriptional changes of Larus ridibundus IRF7 in different groups; **(D)** Transcriptional changes of Larus ridibundus IFN-a in different groups; **(E)** Transcriptional changes of Larus ridibundus Mx in different groups; **(F)** Transcriptional changes of NP gene in different groups. The same uppercase letters indicate no significant difference (*P>0.05*), whereas different uppercase letters indicate extremely significant differences (*P<0.01*). Different lowercase letters indicate significant differences (*0.05<P<0.01*), whereas the same lowercase letters indicate no significant differences (*P>0.05).*.

Lymphocytes receiving the selected NDV dose together with 200 ng/mL IFN-γ had lower NDV NP transcript abundance at 48 and 60 h than lymphocytes infected with NDV alone (*P < 0.01*). Based on the NP transcriptional profile, the 48-h time point was used to compare innate immune pathway-associated genes. At 48 h, TLR7, MyD88, IRF7, IFN-α, and Mx transcript levels in the combined IFN-γ + NDV group were lower than those in both the NDV-only group and the IFN-γ-only group (*P < 0.01*; **Figures 12A–F**). These results show a reduction in the NP transcript endpoint after IFN-γ intervention.

## 4 Discussion

### 4.1 Basic structural characteristics and prokaryotic expression suitability of *Larus ridibundus* IFN-γ

Current research on IFN-γ mainly focuses on mammals and domestic poultry, while studies concerning IFN-γ proteins derived from wild birds remain scarce. In the present study, the CDS of *Larus ridibundus* IFN-γ was cloned into an expression vector to achieve recombinant protein expression. The CDS of *Larus ridibundus* IFN-γ encodes 165 amino acids. Consistent with chicken, duck and other avian species, the molecular weight of *Larus ridibundus* IFN-γ protein is 19.16 kDa. Minor variations in the molecular weight of IFN-γ proteins among different avian species arise from discrepancies in their amino acid sequences. The protein sequence of *Larus ridibundus* IFN-γ contains fewer acidic amino acids and abundant basic amino acids, rendering it sensitive to acidic environments yet tolerant to weakly alkaline conditions. Its high theoretical isoelectric point confirms that this protein belongs to the category of basic proteins. Although *Larus ridibundus* are eukaryotes, the relatively short and evolutionarily conserved sequence of IFN-γ results in only a small number of rare codons without consecutive rare codons, laying a solid foundation for subsequent protein expression in Escherichia coli.

IFN-γ exerts a central and irreplaceable role in the immune system, and its sophisticated immunomodulatory functions have attracted extensive research attention. Nevertheless, the immune-activating and antiviral capacities of *Larus ridibundus* IFN-γ have not been fully elucidated. To address this gap, lymphocytes of *Larus ridibundus* were incubated with serially diluted recombinant IFN-γ protein, and transcriptional levels of immune-related genes were quantified to decipher the regulatory effects of recombinant IFN-γ on target gene transcription in lymphocytes at the transcriptional level.

### 4.2 Dynamic activation characteristics of TLR7/MyD88/IRF7 signaling genes in lymphocytes induced by NDV infection

Lymphocytes were challenged with three NDV doses, and samples collected at 24, 36, 48, and 60 h were analyzed for NDV NP transcripts. In the low-dose group, NP transcription initially increased and then decreased across the 24–60 h interval, with highly significant differences among time points (*P < 0.01*). The medium-dose group showed a similar temporal pattern but higher transcript abundance. The high-dose group showed persistently elevated NP transcription at 48 h, with highly significant differences among sampling times (*P < 0.01*). Comparison among doses at 48 h indicated that this time point was suitable for subsequent temporal monitoring, and the medium viral dose was selected to maintain measurable viral transcription while preserving cell status.

Time-course analysis after *in vitro* stimulation with the selected NDV dose showed that TLR7 and Mx transcripts peaked at 48 h (*P < 0.01*), whereas IRF7 and IFN-α peaked at 36 h. MyD88 transcription increased and then decreased, reaching its maximum at 48 h. IFN-α declined during 48–60 h, while IFN-γ increased continuously from 12 to 60 h (*P < 0.01*). Mx increased from 36 h, peaked at 48 h, and declined at 60 h, with a highly significant difference between 48 and 60 h (*P < 0.01*). These distinct temporal profiles indicate coordinated but nonidentical responses of TLR7 pathway-associated genes to NDV.

These temporal patterns are consistent with transcriptional activation of the TLR7-MyD88-IRF7 axis during NDV infection and with downstream interferon-associated responses. Type I IFN-α predominated earlier in the response, whereas type II IFN-γ showed a cumulative increase across the sampled period. Mx transcription increased later and peaked at 48 h. The coordinated timing supports an association among NDV exposure, pathway-gene transcription, and antiviral effector expression.

### 4.3 Regulatory effects of IFN-γ on the innate immune pathway and their association with anti-NDV activity

The three IFN-γ concentrations produced distinct TLR7 transcriptional patterns. TLR7 transcription increased steadily in the low-dose group and was higher in the medium-dose group (*P < 0.01*). The high-dose group showed a biphasic response, with lower transcription at 60 h than at 48 h (*P < 0.01*). Thus, 200 ng/mL produced the strongest TLR7 transcriptional response among the tested concentrations and was selected for the subsequent cellular antiviral assay. This finding is consistent with reports that IFN-γ can increase TLR7 transcription in human eosinophils and macrophages (Miettinen et al., 2001; Nagase et al., 2003). However, this dose-gradient experiment was limited to detecting the transcription levels of host genes and did not confirm the antiviral effect of IFN-γ.

After treatment of *Larus ridibundus* lymphocytes with medium-dose IFN-γ, the transcription of MyD88, IRF7, IFN-α and Mx increased continuously from 12 h to 60 h, reaching synchronized expression peaks at 48 h. Temporal expression differences of IRF7 were significant (*0.01<P<0.05*), while the other three genes exhibited extremely significant inter-time-point differences (*P<0.01*). When investigating the function of Atlantic salmon MyD88, Skjæveland et al (Skjæveland et al., 2009). reported the level of MyD88 mRNA in TO cells treated with IFN-γ increased; likewise, when IFN-γ binds to CpG oligonucleotides, it can upregulate the transcription of MyD88 in salmon head kidney leukemia cells. Bannon et al (Bannon et al., 2009). explored the regulation of human colonic adenocyte MyD88 expression by human IFN-γ and found that IFN-γ rapidly boosted MyD88 transcription within 2 h and sustained this upregulation until 48 h, with elevated MyD88 protein levels validated at the translational stage. Collectively, these studies prove that IFN-γ modulates MyD88 transcription, consistent with our present results. Zhou et al (Zhou et al., 2016). incubated duck embryo fibroblasts with recombinant goose IFN-γ for 48 h and 60 h, and RT-qPCR detection revealed significant upregulation of IFN-α mRNA relative to untreated controls. After treating DF-1 cells with recombinant chicken IFN-γ, Yang et al (Yang et al., 2020). performed high-throughput RNA sequencing and identified elevated transcription of the Mx1 gene.

In NDV-infected lymphocytes, treatment with 200 ng/mL IFN-γ reduced intracellular NDV NP transcript abundance relative to NDV infection alone (P < 0.01). This result supports *in vitro* anti-NDV activity at the viral-transcript level. TLR7, MyD88, IRF7, IFN-α, and Mx transcripts were also lower in the combined IFN-γ + NDV group than in the NDV-only group (P < 0.01). One possible interpretation is that IFN-γ reduced NDV stimulus and thereby diminished pathway-associated transcription. Since infectious viral titers following IFN-γ intervention were not quantified in the present study, this inference remains to be further validated. The current data only demonstrate a correlative relationship between pathway-related gene transcription and antiviral activity.

### 4.4 Limitations and future directions

Several limitations should be considered. First, all functional assays were performed in primary peripheral blood lymphocytes *in vitro*, the antiviral effect after interferon treatment was inferred primarily from NDV NP transcript abundance. NP protein was assessed only in selected infection experiments, and infectious virus titers were not measured after interferon intervention. Second, involvement of the TLR7/MyD88/IRF7 signaling pathway was inferred from temporal and treatment-associated transcriptional changes. Further experiments employing DF-1 cell models and pathway inhibition assays are required to substantiate the direct mechanistic dependence of IFN-γ function on the TLR7/MyD88/IRF7 signaling pathway.

## 5 Conclusion

The *Larus ridibundus* IFN-γ coding sequence was cloned, recombinant protein was expressed and purified, and specific polyclonal antibodies were generated. NDV infection was associated with temporal changes in TLR7, MyD88, IRF7, IFN-α, IFN-γ, and Mx transcription. Exogenous recombinant IFN-γ also altered the transcription of these genes, and IFN-γ intervention reduced NDV NP transcript abundance in infected lymphocytes. Taken together, the results support *in vitro* anti-NDV activity of *L. ridibundus* IFN-γ and an association with transcriptional modulation of pathway-related genes.

## Data Availability

The original contributions presented in the study are included in the article/supplementary material, further inquiries can be directed to the corresponding author/s.
